# Lipid Metabolism Reprogramming in the Aging Brain: Glial-Mediated Pathogenic Mechanisms and Translational Strategies in Neurodegeneration

**DOI:** 10.3390/ijms27125580

**Published:** 2026-06-20

**Authors:** Wei Shao, Kai Wang, Yongchao Liu, Haojia Zhang, Zijin Sun, Rui Zhou

**Affiliations:** School of Traditional Chinese Medicine, Beijing University of Chinese Medicine, Beijing 102488, China

**Keywords:** cerebral lipid homeostasis, lipid metabolism reprogramming, neurodegenerative diseases, brain aging, apolipoprotein E (APOE)

## Abstract

The mammalian brain fundamentally relies on precise lipid homeostasis to maintain structural integrity and complex neural signaling. Emerging evidence positions lipid metabolism reprogramming not merely as a secondary pathological byproduct but as a core initiating driver of age-related neurodegenerative diseases. This review systematically evaluates the mechanisms of cerebral lipid dyshomeostasis during brain aging, highlighting glial cells as the central mediators of this pathological cascade. We comprehensively dissect the age-associated “lipid drift”, emphasizing apolipoprotein E (APOE)-induced cholesterol transport defects and lipid raft pathology, the accumulation of lipid droplets that triggers microglial metabolic stress (LDAMs), and ceramide-driven neuronal apoptosis coupled with the exosome-mediated propagation of pathogenic proteins. Furthermore, we map these aberrant lipid networks to specific pathological signatures in Alzheimer’s, Parkinson’s, and demyelinating diseases. Finally, we critically evaluate promising therapeutic interventions, including nutritional strategies, LXR/RXR agonists, and nanotechnology-enabled delivery systems designed to bypass the blood–brain barrier. By integrating high-throughput lipidomics for early diagnostic biomarker discovery, we underscore the translational imperative of restoring cerebral lipid homeostasis as a disease-modifying strategy for neurodegeneration.

## 1. Introduction

The brain is a highly lipid-rich organ, with lipids accounting for approximately 50% to 60% of its dry weight [1,2,3]. This complex lipidomic composition includes phospholipids, sphingolipids, cholesterol, glycolipids, and polyunsaturated fatty acids (PUFAs), collectively forming the material foundation of the nervous system [1,4,5]. At the structural level, these lipids not only serve as the core guarantors of cell membrane integrity but also provide the structural framework for myelin formation and synaptic organization [1,3,4]. However, the functions of lipids extend far beyond structural components. They also serve as key signaling molecules and structural integrators, participating in guiding intracellular vesicle transport, regulating enzyme activity, modulating ion channel and transmembrane receptor dynamics, and ultimately influencing synaptic transmission and synaptic plasticity [1,4,6]. Furthermore, lipid distribution within the brain exhibits significant heterogeneity. For instance, the molecular chain length and composition of cardiolipin (CL) in brain tissue markedly differ from those in peripheral organs. This unique lipid profile is crucial for maintaining mitochondrial function and brain homeostasis [7].

**Figure 1:**
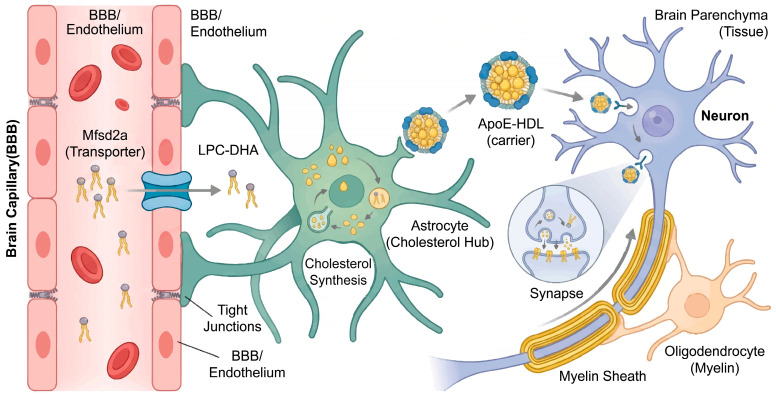
The brain lipid ecosystem: blood–brain barrier and glial–neuronal metabolism. Capillary endothelial cells (red) form the blood–brain barrier (BBB) via tight junctions and facilitate the active transport of LPC-DHA from the capillary lumen into the brain parenchyma via the Mfsd2a transporter. Astrocytes (green) act as the central metabolic hub by extending their end-feet to the BBB, synthesizing cholesterol (yellow droplets) intracellularly, and secreting ApoE-HDL carrier particles (blue/yellow spheres) into the extracellular space. Neurons (blue) subsequently internalize these ApoE-HDL particles via surface receptors to utilize the lipids for cellular maintenance, particularly integrating them into the synaptic membrane (inset). Furthermore, oligodendrocytes (orange) extend processes to tightly wrap neuronal axons, forming the dense, lipid-rich myelin sheath (yellow). Solid grey arrows indicate the predominant direction of lipid flow and carrier transport between cell types, and curved arrows indicate intracellular metabolic and packaging pathways within astrocytes. BBB, blood–brain barrier; LPC-DHA, lysophosphatidylcholine-docosahexaenoic acid; ApoE-HDL, apolipoprotein E-high density lipoprotein; Mfsd2a, major facilitator superfamily domain containing 2A.

The brain exhibits remarkable independence and specificity in lipid metabolism, primarily due to the physical isolation provided by the blood–brain barrier (BBB). The BBB strictly restricts the direct entry of lipids from the peripheral circulation into the central nervous system (CNS), creating a distinct compartmentalization between the brain’s lipid pool and the peripheral pool [2,5,8]. Taking cholesterol as an example, intracellular cholesterol in the brain is primarily synthesized, transported, and recycled through the collaborative actions of neurons and astrocytes, rather than relying on peripheral supply [8,9]. To acquire essential lipid building blocks (such as essential fatty acids), the brain has established a finely tuned regulatory network. It selectively acquires lysophosphatidylcholine or fatty acids from the circulatory system through specialized endothelial gateways (e.g., the choroid plexus and periventricular organs) and specific molecular transporters (e.g., MFSD2A and Tecr), thereby maintaining the BBB’s low permeability [2,10,11]. This dynamic lipid equilibrium system, jointly maintained by neurons, glial cells, and the BBB, is a core prerequisite for sustaining normal neuronal function and neurological health [1,12] (Figure 1).

With the universal increase in global life expectancy, the accelerating aging process has gradually transformed neurodegenerative diseases into a global public health emergency [13,14]. Against the backdrop of an aging society, dementia has become one of the leading causes of death and disability among the elderly [15]. Among these, Alzheimer’s disease (AD), the most prevalent form of dementia, is experiencing a rapid rise in prevalence alongside increasing human lifespan [5,14]. Epidemiological studies indicate that advanced age is a core risk factor for diseases such as AD and Parkinson’s disease (PD) [16,17]. For instance, in in vivo imaging studies of individuals aged 60 to 80, the positron emission tomography (PET) positivity rate for tau protein—reflecting neurodegeneration—significantly increased with age: among amyloid beta (Aβ)-negative cognitively normal individuals, this proportion rose from 1.1% to 4.4%, while in Aβ-positive individuals, it increased from 17.4% to 22.2% [18].

Clinical studies further reveal that the cognitive continuum of AD often begins years or even decades before symptoms manifest. Subjective cognitive decline (SCD) typically occurs 4.4 years before mild cognitive impairment (MCI) and approximately 6.8 years prior to AD diagnosis, indicating a substantial latent disease risk in the aging population [19]. Such an extensive preclinical timeline significantly amplifies the overall public health challenge. Beyond AD, other neurodegenerative diseases also impose a substantial burden in aging populations. Early peripheral pathological changes in PD, such as lipid metabolism disorders, can be detected up to 15 years before clinical diagnosis [20]. Meanwhile, the lifetime incidence risk of amyotrophic lateral sclerosis (ALS) in the general population reaches 1 in 350 [21].

The incidence of neurodegenerative diseases exhibits significant variations across different demographic groups. Gender and ethnicity play crucial moderating roles in disease prevalence patterns, with females typically demonstrating higher AD prevalence rates and accelerated disease progression [16,22]. Similar epidemiological disparities extend to ethnic backgrounds, where certain populations bear a disproportionately higher susceptibility to cognitive decline. This increasing risk is influenced not only by genetic factors (such as the APOE ε4 allele) but also closely linked to modifiable aging-related factors like altered sleep patterns and increased cardiovascular burden [17,23,24].

Maintaining lipid homeostasis in the brain is crucial for the complex functions of the nervous system, particularly for highly specialized synaptic structures and signaling pathways [25,26]. Current academic perspectives are undergoing a significant shift: lipid dyshomeostasis is no longer viewed merely as a secondary phenomenon accompanying aging or neurodegenerative diseases, but rather as a core initiating factor driving neuronal death and glial dysfunction [12,27,28,29]. Pathological lipidome remodeling induces cellular damage through dual physical and chemical mechanisms. For instance, alterations in membrane lipid composition directly modulate γ-secretase processing activity, thereby altering amyloid beta (α–β) production and secretion [28]. Furthermore, failure of the body’s anti-glycation defense systems (such as glyoxalase G10-1) leads to lipidome damage from dicarbonyl stress. This damage directly interferes with cellular signaling and accelerates the progression of aging-related diseases [29].

At the subcellular level, lipid metabolism disorders represent one of the key mechanisms driving neuronal degeneration [30,31,32]. In amyotrophic lateral sclerosis (ALS) and various motor neuron diseases (MNDs), researchers have observed pronounced “subcellular lipidome dysregulation” involving abnormal lipid compositions in specific organelles such as the nuclear envelope, mitochondria, and lysosomes [31,32]. For instance, WDR45 deficiency impairs chaperone-mediated autophagy (CMA), inducing fatty acid synthase (Fasn) accumulation and toxic intracellular lipid droplet (LD) buildup [30]. This localized lipid imbalance is often accompanied by oxidative stress and metal ion homeostasis disruption (e.g., altered phospholipid composition due to iron/manganese exposure), collectively accelerating dopaminergic neuron loss [33].

Glial metabolic reprogramming and the resulting functional impairment represent a critical link in lipid homeostasis disruption driving neurodegeneration [34,35,36,37]. In Alzheimer’s disease models, Aβ pathology induces marked lysosomal lipid accumulation (e.g., BMP and GM3) in cortical and hippocampal microglia, with this lysosomal dysfunction limiting their ability to clear abnormal proteins [34]. App gene knock-in models further confirm that microglia exhibit severe lipid homeostasis disruption after phagocytosing fibrillar Aβ, developing a “foam cell”-like metabolic phenotype [36]. Concurrently, GBA1 inactivation in oligodendrocytes sufficiently disrupts myelin lipid homeostasis to induce demyelination, axonal degeneration, and α-synuclein accumulation [35]. In astrocytes, the APOE4 genotype interacts with sex and the lipid environment to cause abnormal fluctuations in lysosomal calcium signaling and alterations in membrane lipid profiles, thereby disrupting neuronal excitability [37]. These findings collectively demonstrate that initial abnormalities in lipid metabolism trigger multisystem chain reactions ultimately leading to irreversible neurodegeneration.

## 2. Physiological Alterations in Brain Lipid Metabolism During the Aging Process

### 2.1. Drift in Lipid Composition Profile

During physiological brain aging, the lipidome exhibits significant compositional drift, a phenomenon termed “lipid drift” that is recognized as a key precursor to cognitive decline and neurodegenerative diseases [38,39]. Research indicates that although the adult brain possesses efficient mechanisms for maintaining cellular membrane lipid stability, the lipid profiles of the cortex and cerebellum still undergo approximately 10% overall fluctuation upon entering the aging phase [39]. The physiologically aged brain is frequently characterized by a progressive elevation and redistribution of free cholesterol within the neural parenchyma. Comprehensive cell-type-specific tracking across the mammalian lifespan reveals that chronological senescence triggers a fundamental homeostatic shift in glial cholesterol metabolism, primarily driven by a natural decline in the astrocytic cholesterol-synthesis program required for synaptic membrane maintenance [40]. This senescent derangement leads to an aberrant accumulation of unesterified sterols within cell membranes and lipid rafts, profoundly altering glial membrane biophysical dynamics and homeostatic clearance capacity independent of overt neurodegenerative cascades [41]. Moreover, accumulation of the key oxycholesterol 27-hydroxy cholesterol (27-OHC) induces iron overload and mitochondrial dysfunction, driving the “inflammaging” process by triggering microglial senescence [42]. Reduced expression or functional loss of lipid transport receptors (e.g., LSR) further exacerbates cholesterol distribution disorders within the brain [43].

The composition ratio of complex glycosphingolipids and gangliosides to structural phospholipids undergoes dynamic chronological adjustments as an intrinsic feature of biological senescence. A comprehensive untargeted lipidome landscape across lifespan developmental stages demonstrates that physiological aging drives a systemic remodeling of mammalian glycolipid profiles, characterized by the tissue-specific enrichment of structurally altered glycolipid species independently of neurodegenerative disease pathologies [44]. This native senescent drift is orchestrated by the age-dependent metabolic reprogramming of specific biosynthesis enzymes (such as UGT8a), which alters synaptic membrane biophysical properties and represents a fundamental hallmark of the pristine aging lipidome [44]. In white matter regions, the content of myelin-specific lipids (e.g., ceramides and sulfatides) decreases, and myelin lipids exhibit a higher tendency toward saturation due to reduced levels of lipid biosynthetic enzymes [45]. This loss of structural lipids, coupled with altered phospholipid turnover patterns, compromises the microstructural integrity of cell membranes, thereby impairing neuronal orientation and dispersion as well as signal transduction efficiency [46,47].

The decline in long-chain polyunsaturated fatty acids (PUFAs) and impaired lipid acyl chain remodeling constitute another core feature of age-related changes in the brain lipidome. With advancing age, levels of n-3 PUFAs—such as DHA and EPA, which possess neuroprotective and pro-inflammatory resolution effects—significantly decrease in brain tissue [48,49,50]. This PUFA depletion not only arises from dysregulation of lipid acyl chain remodeling pathways (e.g., the Lands cycle) [51] but also correlates closely with age-related evolution of the gut microbiota—the colonization of senile microbiota directly reduces total cortical PUFA content while increasing monounsaturated fatty acids (MUFAs) [50]. PUFA deficiency weakens the brain’s defense against neuroinflammation, promoting the transformation of microglia from a quiescent (M0) state to a reactive neurodegenerative phenotype (MGnD) [52]. Concurrently, environmental factors such as high intake of n-6-dominant PUFAs may further disrupt lipid homeostasis, increasing dementia risk [53]. These systemic shifts in lipid profiles, coupled with abnormalities in peripheral immunometabolic markers (e.g., neutrophil-to-high-density lipoprotein cholesterol ratio), collectively accelerate biological aging in the brain [38,54].

### 2.2. Lipid Peroxidation and “Lipofuscin” Accumulation

Mitochondrial dysfunction during aging is a primary driver of reactive oxygen species (ROS) production [55,56]. In aging-related pathological models or environments affected by specific toxins (e.g., manganese), mitochondrial superoxide production significantly increases, subsequently attacking cell membrane lipids—particularly polyunsaturated fatty acids (PUFAs)—leading to substantial accumulation of iron-dependent lipid peroxides [57,58,59]. Particularly in key genetic contexts like APOE, lipid peroxidation triggered by PUFA-laden lipid particles within lysosomes is considered a critical step in degenerative disease progression [59]. Furthermore, specific environmental pollutants also accelerate oxidative damage to membrane lipids by significantly elevating intracellular ROS levels through suppression of antioxidant enzyme activity [56,60,61].

Lipid peroxidation products fail to be completely degraded within the lysosomes of neurons and microglia, ultimately forming granular substances with spontaneous fluorescence—lipofuscin [60,62]. Lipofuscin accumulation serves not only as a classic biomarker of cellular aging but also functions as a key component of metabolic waste in specific neurodegenerative diseases like CLN2. It co-accumulates with mitochondrial ATP synthase C subunit (SCMAS) and acts as a potential pathological regulator [62,63]. This lipofuscin deposition, resulting from metabolic imbalance, exhibits significant tissue heterogeneity. It primarily occurs in postmitotic cells (e.g., neurons) within the brain’s gray matter regions and may influence cellular inflammatory responses through feedback loops [62].

**Figure 2:**
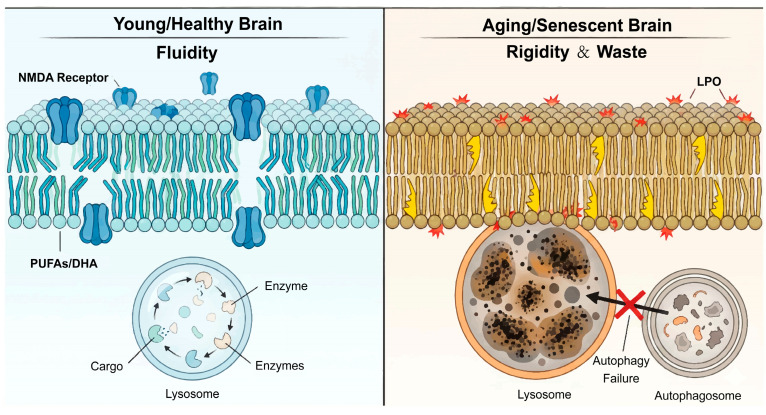
The “drift” of the aging lipidome: membrane rigidity and lipofuscin accumulation. In the young/healthy brain (**left panel**), the cellular membrane exhibits high fluidity, characterized by a phospholipid bilayer rich in “kinked” polyunsaturated fatty acids (e.g., PUFAs/DHA; light blue/green tails), which facilitates the dynamic movement and function of embedded membrane proteins such as NMDA receptors (blue). Intracellularly, healthy lysosomes maintain efficient clearance of cellular cargo via active hydrolytic enzymes. Conversely, in the aging/senescent brain (**right panel**), the membrane lipidome shifts toward increased rigidity and waste accumulation. The bilayer becomes tightly packed with saturated fatty acids (straight brown tails) and intercalated cholesterol molecules (yellow), while the membrane surface exhibits accumulation of lipid peroxidation (LPO) damage (red sparks). Intracellularly, enlarged lysosomes become engorged with undegradable lipofuscin aggregates (dark brown/black granules). This accumulation of pathological waste contributes to widespread autophagy failure, depicted by the blocked fusion (red cross and solid black arrow) between an incoming autophagosome and the lipofuscin-loaded lysosome. Thin black arrows in the young lysosome indicate the normal cyclical process of enzymatic degradation. DHA, docosahexaenoic acid; LPO, lipid peroxidation; NMDA, N-methyl-D-aspartate; PUFAs, polyunsaturated fatty acids.

The persistent accumulation of lipofuscin in lysosomes severely inhibits cellular autophagy, forming a negative feedback mechanism that disrupts homeostasis. Excessive reactive oxygen species can induce lysosomal membrane permeabilization (LMP), leading to lysosomal alkalization and impairing the normal function of lysosomal degradative enzymes (such as cathepsins), thereby blocking autophagic flux [64,65]. Accumulated lipofuscin occupies lysosomal space, diminishing cellular clearance of neurotoxic proteins like tau fibrils and triggering extensive proteomic remodeling [59,62,66]. This functional collapse of the autophagy–lysosomal system not only prevents timely clearance of damaged mitochondria via mitophagy but also further induces bursts of intracellular oxidative stress, ultimately accelerating cellular senescence and driving neurodegeneration [56,62,64,65] (Figure 2).

### 2.3. Loss of Myelin Integrity

The health of brain white matter is highly dependent on the sustained maintenance of myelin by oligodendrocytes (OLs), a process with extremely high energy demands [67]. To sustain this continuous myelin remodeling, OLs must maintain a robust and delicately balanced metabolic network under normal physiological conditions. However, with brain aging, mature postmitotic oligodendrocytes enter a state of cellular senescence (PoMiCS) through activation of the IKK/NF-κB signaling pathway. This leads to decreased myelin protein expression and disrupted myelin homeostasis, ultimately triggering characteristic white matter rarefaction [67]. Research indicates that this loss of remyelination capacity and significant suppression of myelin renewal constitute one of the core pathological mechanisms underlying spatial memory deficits in aging individuals [68,69].

The interplay between structural damage and chronic inflammation further exacerbates the loss of white matter integrity. Spatial transcriptomics studies reveal that white matter tracts in aging brains serve as focal points for inflammatory responses, characterized by abnormal microglial activation, complement activation, and myeloid cell infiltration [70]. This maladaptive immune response recruits pathogenic CD8+ T cells, directly leading to myelinated axonal degeneration [71]. In neurodegenerative diseases like Alzheimer’s, loss of myelin integrity is even considered an upstream driver of Aβ deposition: myelin dysfunction causes the Aβ production machinery to accumulate at axonal swellings and distracts microglia—which should be clearing plaques—toward addressing nearby myelin damage [72]. Furthermore, elevated aerobic glycolysis and oxidative phospholipid deposition in white matter are closely associated with exacerbated white matter injury [73,74].

From a functional perspective, pathological demyelination directly leads to a significant reduction in nerve conduction velocity, representing a key electrophysiological hallmark of age-related cognitive decline [75,76]. Clinically, this manifests as increased white matter hyperintensity (WMH) burden and diminished microstructural integrity metrics (e.g., graded anisotropy), changes highly correlated with individual declines in executive function, processing speed, and overall cognitive capacity [77,78,79]. Addressing this crisis, interventions such as inducing epigenetic remodeling with small-molecule inhibitors (e.g., ESI1) to activate SREBP-mediated lipid biosynthesis or employing intermittent fasting have been demonstrated to effectively promote myelin regeneration and reverse age-related cognitive decline [79,80,81].

## 3. Cholesterol Metabolic Disorders: From ApoE to Amyloid Plaques

### 3.1. Transport of Cerebral Cholesterol and ApoE Genotype

In the brain, maintaining cholesterol homeostasis is crucial for synaptic formation, maintenance, and complex neural functions [82,83]. Due to the physical isolation provided by the blood–brain barrier, the brain cannot directly acquire cholesterol from the peripheral circulation. Its metabolism exhibits a high degree of independence, primarily relying on in situ synthesis by astrocytes and other glial cells [82,84]. Synthesized cholesterol is encapsulated within HDL-like particles, primarily composed of apolipoprotein E (ApoE), and distributed via astrocyte-to-neuron supply pathways to support neuronal energy metabolism and synaptic plasticity [82,85].

As the strongest genetic risk factor for late-onset Alzheimer’s disease (LOAD), the APOE4 genotype exhibits significant defects in lipid binding and transport efficiency [82,86,87]. Compared to ApoE2 or ApoE3, the ApoE4 protein demonstrates markedly reduced lipid binding efficiency due to structural alterations caused by a single amino acid difference [88,89]. Experimental studies reveal that APOE3 astrocytes generate larger, more lipid-rich HDL particles, effectively rescuing neuronal lipid abnormalities and dystrophic neurites. Conversely, APOE4 impairs cholesterol efflux, inducing cholesterol overload, lipid droplet (LD) accumulation, and lysosomal dysfunction in neurons and glial cells [88,90,91,92]. This impaired transport capacity ultimately results in pathological cholesterol deficiency within neurons, compromising normal synaptic development and signaling [84,93].

APOE4-mediated cholesterol metabolism disorders further disrupt metabolic coupling between astrocytes and neurons, directly impairing synaptic plasticity and disrupting neural network activity [85,94]. The ApoE4 protein not only interferes with LRP1 receptor function and inhibits insulin signaling but also exacerbates tau phosphorylation by regulating GSK3β activity [86]. Multi-omics analyses reveal that neurons from APOE4 carriers exhibit marked metabolic stress and mitochondrial dysfunction, along with abnormal neuronal excitability under inflammatory conditions [94,95]. Furthermore, ApoE4-induced alterations in membrane lipid composition (e.g., increased ceramides) and disrupted lipid homeostasis not only diminish astrocytic synaptic support for neurons but also exacerbate neurotoxicity by activating inflammatory pathways (e.g., TLR4/NF-κB), creating a vicious cycle that drives neurodegeneration [85,88].

### 3.2. Lipid Rafts and Protease Cleavage

Lipid rafts are liquid-ordered microdomains on neuronal cell membranes enriched in cholesterol and sphingolipids, recognized as the core site for amyloid precursor protein (APP) processing that triggers amyloidogenesis [96,97,98]. Structurally analogous to type I membrane receptors, the APP functions as a cholesterol sensor within lipid rafts. Its subcellular localization, dimerization of transmembrane domains, and interaction with secretory enzymes are highly dependent on the local lipid environment [96,99,100]. Studies indicate that β-secretase (BACE1) and γ-secretase complex components (such as presenilin 1) preferentially localize to lipid rafts [97,101,102]. Specifically, palmitoylated APP (palAPP) migrates preferentially to lipid rafts and mitochondria-associated membrane (MAM) surfaces, where it serves as a preferred substrate for BACE1 to initiate the Aβ generation cascade [103].

Abnormal cholesterol accumulation serves as the initiating factor driving excessive Aβ production. APOE4-positive astrocytes induce physical expansion of neuronal lipid rafts through excessive cholesterol secretion, thereby providing a larger platform for APP and BACE1 aggregation and promoting Aβ42 synthesis [104,105]. Furthermore, metabolic stress or inflammatory signals can further disrupt this process: for example, SERP1 protein under metabolic stress enhances γ-secretase assembly and localization within lipid rafts [106]; circulating complement C1q can upregulate BACE1 expression and activity within lipid rafts by activating the JAK2-STAT1 pathway [107]. Although some studies suggest that reduced cholesterol content in detergent-resistant membranes (DRMs) from sporadic AD patients may enhance Aβ production [102], the prevailing theory maintains that cholesterol-mediated liquid–liquid partitioning serves as a critical switch regulating APP endocytosis and entry into the pro-amyloid pathway [97,100,101].

**Figure 3:**
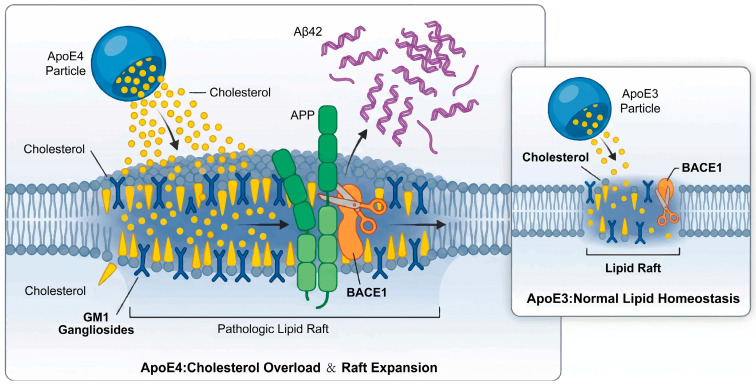
ApoE4 and lipid raft pathology: the “lipid bed” for amyloid generation. In the pathological state (**main panel**), the ApoE4 particle (blue) aberrantly delivers an excessive load of cholesterol (yellow) to the neuronal plasma membrane. This cholesterol overload drives the massive expansion of the lipid raft domain, rendering it structurally thickened and densely packed with intercalated cholesterol and GM1 gangliosides (dark blue). This expanded “lipid bed” facilitates the pathological spatial clustering of the transmembrane amyloid precursor protein (APP; green) and the beta-secretase enzyme BACE1 (orange). Their forced proximity within this expanded microdomain promotes active BACE1-mediated cleavage of APP, resulting in the robust generation and extracellular accumulation of neurotoxic amyloid beta 42 (Aβ42) fibrils (purple). Conversely, under normal physiological conditions (**inset**), the ApoE3 particle maintains cellular lipid homeostasis by delivering regulated levels of cholesterol. This supports a small, discrete lipid raft where APP and BACE1 remain physically segregated, effectively minimizing aberrant Aβ production. Solid black arrows indicate the influx of cholesterol, the lateral clustering movement of membrane proteins within the raft, and the extracellular release of Aβ42. Aβ42, amyloid beta 42; ApoE, apolipoprotein E; APP, amyloid precursor protein; BACE1, beta-site amyloid precursor protein cleaving enzyme 1; GM1, monosialotetrahexosylganglioside.

Lipid rafts not only regulate Aβ production but also mediate its neurotoxicity and inflammatory feedback loops. Aβ oligomers induce microglia to form enlarged, cholesterol-rich “inflammatory rafts”, which serve as assembly platforms for inflammatory receptors such as TLR4, CD36, and TREM2, amplifying neuroinflammatory signaling [108,109,110]. At the synaptic level, Aβ causes GM1 ganglioside accumulation within rafts, inducing abnormal internalization of the glutamate receptor GluA1 and impairing long-term potentiation (LTP) effects [111]. Furthermore, oxidative damage and increased ceramides disrupt the physiological order of lipid rafts. This not only alters the cleavage precision of γ-secretase to generate more toxic Aβ42 subtypes but also promotes the formation of calcium-permeable amyloid pores, ultimately accelerating neuronal disintegration [112,113] (Figure 3).

### 3.3. Neurotoxicity of Oxysterols

In the dynamic equilibrium of brain cholesterol, oxysterols play a dual role as both metabolic “safety valves” and pathological “toxic molecules”. Due to the cytotoxicity of excess free cholesterol, the brain primarily relies on neuron-specific expression of cholesterol 24S-hydroxylase (CYP46A1) to convert cholesterol into 24S-hydroxycholesterol (24S-OHC/24HC) [114,115]. 24S-OHC serves as the primary pathway for cholesterol efflux from the brain. Due to its enhanced membrane permeability, it can cross the BBB into peripheral circulation [115]. However, this clearance mechanism may become dysregulated with aging; elevated levels of CYP46A1 and 24S-OHC have been observed in cerebrospinal fluid from PD patients and in aged mouse models [114].

Although 24S-OHC is a physiologically cleared metabolite, its abnormal accumulation induces severe neurotoxicity. Mechanistically, 24S-OHC has been demonstrated to be a potent allosteric modulator of the glutamate N-methyl-D-aspartate receptor (NMDAR), and its elevated levels cause neuronal hyperexcitability and excitotoxicity [116,117,118]. Furthermore, 24S-OHC exacerbates the pathological evolution of α-synuclein (α-Syn) by disrupting mitochondrial function and activating the XBP1-LAG3 axis, thereby promoting the spread of pathogenic proteins in the brain and the degeneration of dopaminergic neurons [114]. Therefore, controlling 24S-OHC-mediated excitotoxicity by inhibiting CYP46A1 activity (e.g., using soticlestat or glycyrrhizin polysaccharides) has emerged as a novel therapeutic strategy for epilepsy and related neurodegenerative diseases [116,117,118].

Meanwhile, the role of another oxygenated cholesterol—27-OHC—in aging and related pathologies should not be overlooked. 27-OHC typically enters the brain via the BBB from the circulatory system or accumulates intracranially when metabolic enzymes such as CYP7B1 are deficient [119,120]. 27-OHC is widely recognized for its potent neurotoxicity. It not only disrupts BBB integrity but also disturbs the Th17/Treg immune balance, inducing pro-inflammatory cytokine release and thereby impairing learning and memory capabilities [120,121]. Research indicates that while 24S-OHC can antagonize the neurotoxicity of 27-OHC at certain concentrations, the combined dysregulation of these oxysterols against the backdrop of age-related lipid clearance impairment collectively constitutes the molecular basis for cognitive decline [121].

## 4. Fatty Acids and Lipid Droplets: Metabolic Stress in Glial Cells

### 4.1. Abnormal Accumulation of Lipid Droplets (LDs)

In the brain microenvironment of aging and neurodegenerative diseases, microglia exhibit significant metabolic heterogeneity, with one key pathological subpopulation defined as lipid droplet-accumulating microglia (LDAM) [122,123,124]. LDAMs predominantly accumulate in aging white matter regions, characterized by massive intracellular LD accumulation, impaired phagocytic capacity, elevated ROS levels, and robust pro-inflammatory factor release [122,124,125]. The transcriptional profile of these cells is driven by innate immune inflammation, functionally distinct from classical disease-associated microglia (DAM) or neurodegenerative phenotypes (MGnD), representing a highly dysfunctional inflammatory state [122,123,124].

The pathological accumulation of lipid droplets within microglia results from the combined effects of excessive lipid uptake and impaired degradation. During white matter ischemia or myelin injury, microglia actively phagocytose lipid-rich myelin debris. However, age-related dysfunction of the autophagy–lysosomal pathway—such as lysosomal alkalization or impaired lipophagocytosis—prevents efficient degradation or efflux of these lipids, ultimately leading to their storage as triglycerides (TGs) within lipid droplets [122,126,127,128,129]. Molecular mechanism studies reveal that hyperacetylation of fatty acid synthase (FASN) [130], the conversion of free fatty acids to triglycerides mediated by diacylglycerol O-acyltransferase 2 (DGAT2) [127], and abnormal expression of enzymes such as ACSL1 are key drivers of lipid droplet formation [131]. Furthermore, genetic risk factors such as APOE4, PICALM, or GRN deficiency exacerbate lipid metabolism disorders, inducing microglial transformation into “foam cells” resembling those in atherosclerosis [125,127,129,132].

The excessive accumulation of LDs is not only a manifestation of metabolic stress but also a trigger for chronic neuroinflammation. LDAMs continuously release proinflammatory cytokines and chemokines such as TNF-α, IL-1β, IL-6, and IL-18. This proinflammatory phenotype is amplified through metabolic-inflammatory axes like NF-κB-SREBP1 [131,132,133]. This pathological state creates a vicious cycle: lipid-overloaded microglia lose their ability to clear amyloid beta (Aβ) and pathological synapses, while their secreted toxic factors further induce tau phosphorylation, neuronal excitability abnormalities, and impaired network function [124,127,131,132,133]. Studies indicate that pharmacologically activating the TRPV1-PKM2 axis, inhibiting DGAT2, or clearing senescent cells can reduce lipid droplet accumulation and reverse the inflammatory phenotype of microglia, thereby improving age-related cognitive impairment [127,133].

### 4.2. Fatty Acid Oxidation (FAO) Impairment

Astrocytes serve as the primary site for fatty acid oxidation (FAO) in the CNS. Under physiological conditions, ketone bodies produced by astrocytes via FAO constitute a crucial oxidative fuel source for neurons in glucose-restricted environments [134,135]. However, in aging and neurodegenerative diseases such as AD and ALS, astrocytes commonly exhibit bioenergetic depletion and mitochondrial dysfunction [134,136]. Research indicates that defects in mitochondrial oxidative phosphorylation (OxPhos) are a key driver of abnormal lipid accumulation within astrocytes [137]. This diminished FAO capacity prevents free fatty acids (FFAs) from entering mitochondria for efficient metabolism, compelling cells to sequester excess fatty acids by converting them into LDs [134,138,139].

FAO-induced lipotoxicity is a core mechanism leading to astrocyte dysfunction and phenotypic switching. When FAO is impaired, accumulated FFAs are further converted into long-chain acylcarnitines (LCACs) or ceramides. These metabolites induce mitochondrial membrane permeabilization, releasing mitochondrial DNA (mtDNA) into the cytoplasm and activating either the cGAS/STING pathway or NF-κB signaling [134,139,140]. Taking the saturated fatty acid palmitic acid (PA) as an example, the lipotoxicity it induces disrupts the autophagy–lysosomal system, triggers oxidative stress, and induces the release of proinflammatory cytokines (such as IL-1β and TNF-α), transforming astrocytes into a reactive phenotype toxic to neurons (e.g., LARA subtype) [137,141].

**Figure 4:**
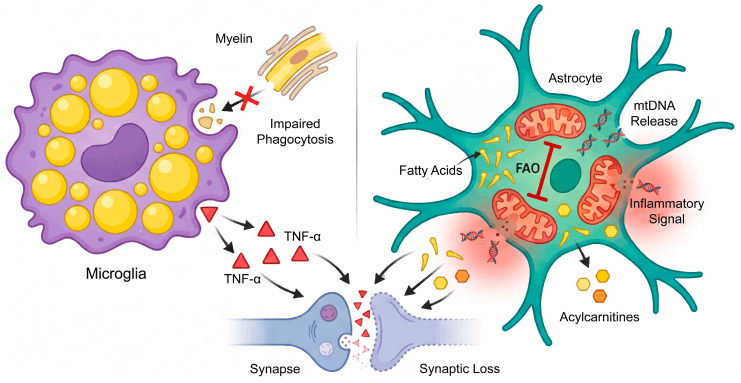
Glial metabolic reprogramming: LDAM and astrocyte lipotoxicity. In the pathological microenvironment, glial cells undergo distinct metabolic maladaptations that collectively drive neurodegeneration. Microglia (purple, **left**) transition into an amoeboid, lipid-droplet-accumulating microglia (LDAM) phenotype, characterized by the massive cytosolic accumulation of lipid droplets (yellow spheres). This state induces profound functional deficits, notably the impairment of myelin debris phagocytosis (red cross), and promotes the robust secretion of pro-inflammatory cytokines, including TNF-α (red triangles). Simultaneously, astrocytes (teal, **right**) experience severe lipotoxicity and mitochondrial stress (red). A metabolic blockade in fatty acid oxidation (FAO; red T-bar) leads to the pathological accumulation of unmetabolized fatty acids (yellow tails) and acylcarnitines (orange hexagons). Consequent mitochondrial damage results in the cytosolic release of mitochondrial DNA (mtDNA), which acts as a danger signal to ignite intracellular inflammatory cascades (red glow). The synergistic onslaught of microglia-derived TNF-α and astrocyte-derived toxic lipid mediators ultimately targets the neuronal synapse (blue, **bottom**), precipitating structural degeneration and profound synaptic loss (fading/dotted borders). Solid black arrows indicate the secretion and directional targeting of inflammatory factors and toxic metabolites. FAO, fatty acid oxidation; LDAM, lipid-droplet-accumulating microglia; mtDNA, mitochondrial DNA; TNF-α, tumor necrosis factor alpha.

Furthermore, the diminished FAO capacity of astrocytes directly disrupts the metabolic coupling between astrocytes and neurons. As impaired FAO is accompanied by glycolytic pathway remodeling, the crucial lactate shuttling mechanism is weakened. This deprives neurons of metabolic support, inducing a chronic energy stress state that exacerbates Aβ deposition, tau phosphorylation, and impaired synaptic plasticity [136,142]. Targeting this pathological pathway, enhancing PPARα-mediated fatty acid oxidation, reversing LD accumulation with lactate dehydrogenase (LDH) inhibitors, or promoting lipid mobilization by enhancing lipid droplet-mitochondrial contact via drugs (e.g., alimorol) has been demonstrated to effectively mitigate lipotoxicity damage and improve cognitive and motor function in AD or ALS models [134,138,143] (Figure 4).

### 4.3. Phospholipid Metabolism and Membrane Fluidity

The biophysical properties of neuronal cell membranes are largely determined by their phospholipid composition, particularly the abundance of long-chain PUFAs [144,145]. Docosahexaenoic acid (DHA, 22:6, n-3), as the most abundant n-3 PUFA in the brain, is a core component of cell membrane phospholipids and is crucial for maintaining membrane integrity, fluidity, and synaptic plasticity [144,145]. Under physiological conditions, DHA readily integrates into membrane phospholipids, regulating lipid packing to maintain optimal membrane environments [146,147]. However, with aging, the brain exhibits pronounced “lipid raft aging”: n-3 LCPUFA content significantly declines, while saturated fatty acids (SFAs) and sterol ester levels correspondingly increase [148,149]. This compositional shift directly leads to increased membrane microviscosity, i.e., a marked reduction in membrane fluidity [148].

Reduced membrane fluidity and disruption of the lipid microenvironment profoundly impact the function of membrane proteins, particularly neurotransmitter receptors. Studies indicate that age-related lipid raft reorganization alters the distribution ratio and interactions between ionotropic and metabotropic glutamate receptors (such as NMDA receptors, AMPA receptors, and mGluR5) within lipid raft and non-raft compartments [148]. This structural redistribution directly impairs receptor responsiveness to signals, thereby compromising long-term potentiation (LTP) effects, and spatial learning and memory capabilities [145,148]. Furthermore, increased membrane lipid accumulation further disrupts intracellular signaling and inter-organelle communication by limiting interactions between biomolecular aggregates and the membrane [146].

For synaptic dysfunction caused by phospholipid metabolism disorders, restoring membrane fluidity through nutritional intervention demonstrates significant therapeutic potential. Supplementation with DHA-rich phosphatidylserine (PS) or n-3 PUFA from fish oil effectively reduces membrane microviscosity in aged brains, restoring it to youthful levels. This promotes the re-enrichment of receptors like NMDA within lipid rafts, thereby reversing the decline in synaptic plasticity [148,150]. Experimental evidence further indicates that DHA supplementation prevents neurotransmission disorders. By optimizing synaptic membrane organization and enhancing antioxidant defenses, it delays or even reverses age-related cognitive decline [144,147,151].

### 4.4. Peripheral–Central Crosstalk: Metabolic Syndrome and Eicosanoid-Driven Neuroinflammation

Type 2 diabetes and obesity, among other metabolic syndromes, are not only indicators of peripheral metabolic disorders but also significant upstream risk factors for AD and related neurodegenerative diseases. Their pathological mechanisms are largely characterized by highly overlapping insulin resistance and lipid infiltration [152,153]. Long-term high-fat diet (HFD), a core driver of metabolic syndrome, can significantly disrupt lipid homeostasis in the central nervous system. Under prolonged exposure to HFD, the metabolic pathways of unsaturated fatty acids such as arachidonic acid (AA) in the cerebral cortex and hypothalamus are abnormally activated, leading to early neuroinflammation and pathological accumulation of a large amount of pro-inflammatory eicosanoids and other oxidized lipids [154,155,156]. Additionally, poor dietary structures like HFD can significantly reduce the content of plant sterols and other lipids with natural anti-inflammatory effects in the brain, thereby removing the inhibition on pro-inflammatory lipid mediators such as prostaglandin D2 (PGD2) and thromboxane B2, further deteriorating the microenvironment within the brain [157].

Pro-inflammatory eicosanoids play a key role in the vicious cycle that links metabolic stress with central nervous system inflammaging [158,159]. The abnormal expression of phospholipase A2 (PLA2), as the upstream rate-limiting enzyme in this cascade, in the ventromedial hypothalamus directly drives the excessive production of prostaglandins, thereby inducing hyperphagia and obesity and disrupting energy metabolism balance [160]. At the specific lipid mediator level, prostaglandin E2 (PGE2) can activate hypothalamic melanin-concentrating hormone (MCH) neurons through a bidirectional concentration-dependent mechanism, exacerbating diet-induced obesity (DIO) and metabolic disorders [161]; meanwhile, the PGE2 receptor EP4 on the surface of microglia, upon sensing high-fat signals, significantly alters the phagocytic state of microglia and enhances the host’s susceptibility to diet-induced insulin resistance [162]. Besides PGE2, excessive PGD2 can also strongly activate microglia and induce a reduction in neurogenesis, hippocampal atrophy, and cognitive decline [163]. Additionally, other polyunsaturated fatty acid derivatives such as 20-HETE, under pathological conditions, can also exacerbate brain tissue damage by inducing oxidative endothelial injury and neuroinflammatory pathways [164].

This diet-mediated peripheral metabolic disorder not only triggers a prostaglandin storm but also induces metabolic endotoxemia and leads to severe dysregulation of the endocannabinoid system (such as AEA and 2-AG). Metabolic stress causes disordered expression of cannabinoid receptors (CB1/CB2), exacerbating microglial activation and synaptic toxicity in the aging brain, ultimately manifesting as high anxiety-like behavior and cognitive dysfunction [165,166]. In neuropathological models with concurrent diabetes, this lipid metabolic network imbalance is also characterized by abnormally elevated pro-inflammatory leukotriene B4 (LTB4) and impaired synthesis of pro-resolving mediators (SPMs), locking macrophages/microglia in the M1 pro-inflammatory phenotype and preventing the initiation of the key resolution program of neuroinflammation [167].

## 5. Sphingolipid Metabolism: The Lethal Signal of Ceramides

### 5.1. Ceramide: Messenger of Cell Death

Sphingolipid metabolites, particularly ceramides, are widely recognized as key signaling molecules that trigger apoptosis in the progression of neurodegenerative diseases [168,169]. In aging brains and pathological environments such as AD, PD, and multiple sclerosis (MS), ceramide levels exhibit a significant and widespread upward trend due to hyperactive acid sphingomyelinase (ASM) activity or abnormalities in de novo synthesis pathways [168,169,170]. Research indicates that accumulation of these “lethal lipids” extends beyond the central nervous system. Elevated levels of specific ceramide species (e.g., C16:0 and C18:1) are observed in pathological cerebrospinal fluid (CSF) and peripheral circulation, with concentrations correlating closely to cognitive impairment severity and neuronal death risk [168,169,171].

The core mechanism by which ceramides mediate neuronal apoptosis lies in their direct disruption of mitochondrial outer membrane integrity [103,140]. Accumulated ceramides (including ultra-long-chain 1-deoxy-dihydroceramides) possess the ability to penetrate and remodel biological membranes, inducing the formation of the mitochondrial permeability transition pore (mPTP) and activating the pro-apoptotic protein BAX [172,173]. For example, C16 ceramides generated by CerS6 synthase induce excessive mitochondrial fission and functional collapse by binding to mitochondrial fission factor (Mff) [173]. This alteration in membrane permeability leads to the release of cytochrome C and mtDNA into the cytoplasm, triggering the caspase cascade and cGAS/STING-mediated inflammatory signaling, ultimately resulting in irreversible programmed neuronal death [140,173].

Additionally, ceramides further amplify apoptotic signals by disrupting the homeostasis of mitochondrial-associated endoplasmic reticulum membranes (MAMs). They displace sphingomyelin and cholesterol from the MAM region, impairing mitochondrial oxidative phosphorylation and inhibiting fatty acid β-oxidation, thereby trapping neurons in a state of “virtual hypoglycemia” characterized by energy depletion [136,173,174]. This metabolic stress is frequently accompanied by impaired autophagy flux (e.g., ceramide-mediated disruption of p62-mediated mitochondrial autophagy) and lysosomal membrane permeabilization (LMP), compromising cellular clearance of pathogenic proteins such as Aβ and α-synuclein [169,171,175]. This ceramide-driven “mitochondrial–lysosomal” dual assault synergistically induces microglial pyroptosis and massive neuronal loss, constituting the terminal lethal link in the “lipid-life crisis” of the aging brain [168,174].

### 5.2. Sphingomyelinase and Exosome Transmission

**Figure 5:**
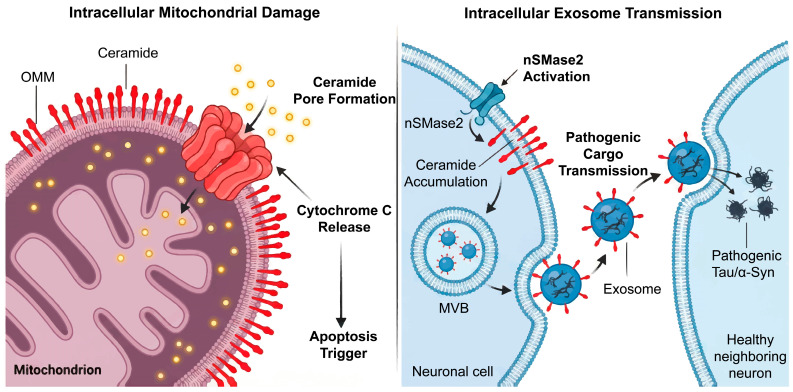
Ceramide’s lethal mechanisms: mitochondrial pores and exosome propagation. (**Left**) Intracellular mitochondrial damage is driven by the robust accumulation of ceramide (red lipids) on the outer mitochondrial membrane (OMM). These sphingolipids self-assemble to form large ceramide pores, physically compromising membrane integrity. This permeabilization facilitates the rapid efflux of cytochrome c (yellow dots) from the intermembrane space into the cytosol, serving as the primary trigger for the apoptotic cascade. (**Right**) Concurrently, ceramide facilitates the intercellular propagation of toxic proteins. The activation of neutral sphingomyelinase 2 (nSMase2) at the neuronal plasma membrane catalyzes local ceramide generation, which drives inward membrane invagination and the biogenesis of multivesicular bodies (MVBs). These intracellular compartments effectively package early-stage toxic protein species, such as soluble or oligomeric pathogenic Tau or α-synuclein (α-Syn; dark grey fibrils), into exosomes. This exosomal release represents an active early-stage mechanism prior to the formation of dense intracellular tangles and subsequent neuronal death. Following membrane fusion, these cargo-loaded exosomes are released into the extracellular space and subsequently internalize into healthy neighboring neurons, mediating the transmission of neurodegenerative pathology. Solid black arrows indicate the sequence of biochemical events and directional transport. MVB, multivesicular body; nSMase2, neutral sphingomyelinase 2; OMM, outer mitochondrial membrane; α-Syn, alpha-synuclein.

Abnormal activation of sphingolipid metabolism enzymes constitutes a core pathological mechanism driving the dissemination of pathogenic proteins within the brain via exosomes/EVs. Research indicates that sphingomyelin hydrolysis mediated by neutral sphingomyelinase 2 (nSMase2) serves as a critical switch triggering EV biosynthesis. In AD models, expression of human tau protein significantly upregulates nSMase2 activity, leading to localized ceramide accumulation in cell membranes. This induces massive formation and extracellular release of EVs carrying phosphorylated tau (pTau) [176]. These EVs, derived from neurons and microglia, act as carriers that propagate the pathogenic protein across different brain regions along synaptically connected networks. The diffusion rate of tau protein positively correlates with intracerebral ceramide levels and nSMase2 activity [176,177] (Figure 5).

A similar “lethal packaging” mechanism exists in Lewy body disease (LBD). Regardless of GBA1 mutation status, brain tissue and CSF from LBD patients exhibit marked sphingolipid metabolism disorders and elevated ceramide levels [178]. Ceramide-rich EVs not only carry substantial amounts of α-synuclein (α-syn) and tau proteins, but their membrane lipid components can also directly interact with α-syn monomers, lowering the aggregation threshold and inducing pathological aggregation and fibril formation in wild-type α-syn [178,179]. Furthermore, gangliosides (e.g., GM1), as key members of the sphingolipid family, also participate in EV biosynthesis by regulating membrane properties. They enhance the exclusion and transport of misfolded proteins (e.g., mHTT, α-syn, and Tau) by EVs, reflecting the dual regulatory role of the sphingolipid system in the extracellular clearance and propagation of pathogenic proteins [180].

Crucially, it is imperative to distinguish the temporal stages of these propagation mechanisms. The exosome-mediated pathway primarily represents an early-stage cellular response. During this phase, before the formation of mature, densely packed neurofibrillary tangles (NFTs), neurons actively utilize the ceramide-dependent exosomal sorting machinery to read-off and expel soluble or oligomeric forms of abnormal proteins. Conversely, in the late stages of the disease, the massive intracellular accumulation of highly packed pTau aggregates disrupts the cytoplasmic architecture, ultimately culminating in neuronal death. Upon cellular lysis, these dense tau tangles and associated lipid membrane debris are passively released into the extracellular space. This late-stage event not only robustly activates surrounding microglia but also facilitates direct cell-to-cell propagation, as the released tau seeds utilize heparan sulfate proteoglycans (HSPGs) on the surface of adjacent healthy neurons to penetrate and propagate the pathology [181,182].

This exosome-mediated “prion-like” propagation pattern serves as a key driver of progressive neurodegeneration in neurodegenerative diseases. Exosomes carrying oxidative stress signals, proinflammatory factors (such as S-nitrosylated proteins), and pathogenic protein fragments induce mitochondrial dysfunction and protein homeostasis imbalance in recipient cells after distant uptake [176,183,184]. Pharmacological inhibition of the nSMase2 pathway (e.g., using PDDC inhibitors) has demonstrated significant therapeutic potential against this process: by correcting abnormal sphingolipid metabolism, it not only reduces the encapsulation of pathological proteins like pTau but also effectively blocks the spread of pathogenic proteins to brain regions such as the contralateral hippocampus. This protects synaptic function and alleviates cognitive decline [176,185].

## 6. Disease-Specific Lipid Pathology

### 6.1. Alzheimer’s Disease

The pathological progression of AD lies at the intersection of lipid metabolism and immune responses, with cholesterol homeostasis imbalance recognized as a core driver of disease progression [186,187]. As the primary lipid transporter in the brain, the APOE4 of ApoE represents the strongest genetic risk factor for late-onset AD [14,187]. Research indicates that APOE4 induces intracellular lipid homeostasis disruption through multiple pathways. In glial cells derived from human-induced pluripotent stem cells (iPSCs), APOE4 leads to increased fatty acid unsaturation and abnormal accumulation of cytoplasmic LDs. Additionally, increased expression of non-vesicular cholesterol transporter subtypes (e.g., GRAMD1B) and inhibition of the cholesterol clearance enzyme CYP46A1 by the oligomerized mitochondrial AAA-ATPase ATAD3A collectively lead to intracellular free cholesterol accumulation in neurons, thereby promoting amyloid precursor protein (APP) processing and synaptic loss [188,189]. In contrast, protective variants (e.g., ApoE2 and ApoE3 Christchurch) facilitate efficient efflux of oxidized unsaturated lipids via the ABCA7 transporter, thereby mitigating endoliposomal dysfunction and excitotoxicity in neurons [190].

The compromised integrity of the BBB represents an early pathological feature in APOE4 carriers, often occurring independently of Aβ or tau pathology [191]. APOE4 derived from astrocytes activates the cyclophilin A-matrix metalloproteinase-9 (MMP-9) pathway in pericytes, leading to tight junction protein degradation and microvascular leakage [191]. This compromised barrier function not only impedes intracerebral waste clearance but also facilitates leakage of peripheral proinflammatory factors and blood proteins (e.g., fibrinogen), further inducing glial polarization toward a neurotoxic phenotype [192,193]. Concurrently, APOE4 impairs the metabolic function of brain microvascular endothelial cells (BMECs), manifested by reduced SIRT1 levels and weakened insulin signaling, thereby exacerbating hypoglycaemic metabolism in the brain [194].

Impaired lipid transport further exacerbates AD pathological changes through multicellular feedback loops. At the blood–brain barrier level, lysoPCs—as key carriers for transporting neuroprotective polyunsaturated fatty acids into the brain—exhibit reduced plasma levels closely associated with amyloid pathology in APOE4 carriers [195]. Beyond the vascular interface, localized lipid recycling within the brain parenchyma is equally disrupted, particularly concerning the clearance of lipid-dense cellular waste. Furthermore, TREM2-deficient or dysfunctional microglia fail to efficiently clear cholesterol from myelin debris, leading to intracellular cholesterol ester (CE) accumulation and loss of phagocytic capacity against Aβ plaques [196,197]. Intervention studies targeting this pathway demonstrate that pharmacologically enhancing cholesterol transport—such as activating LXRs or using TRPV1 agonists to reduce lysosomal cholesterol accumulation—effectively reduces Aβ deposition and improves cognitive impairment in AD models [198].

### 6.2. Parkinson’s Disease

The pathological aggregation of α-synuclein (αSyn) and its progression into Lewy bodies represent the core pathological hallmarks of PD, a process closely associated with interactions with neuronal lipid membranes [199,200]. αSyn itself is a lipid-binding protein that, under physiological conditions, binds to synaptic vesicle membranes via an amphipathic helix structure, participating in vesicle anchoring and fusion [179,199]. However, alterations in lipid composition significantly impact αSyn’s conformational stability: negatively charged lipids (e.g., phosphatidylglycerol) induce β-folding transitions and promote fibrillation, whereas neutral phosphatidylcholine (PC) tends to inhibit aggregation [179,199]. Furthermore, reduced membrane fluidity and peroxidation of PUFAs induce abnormal calcium influx by altering membrane conductance. Acting as scaffolds, these changes accelerate β-sheet protein incorporation at the membrane surface, ultimately driving ferroptosis and neuronal apoptosis [200].

Defective function of glucosylceramidase (GCase, encoded by the GBA1 gene) is the most significant genetic factor causing lipid metabolism disorders in PD [201,202]. GCase is responsible for degrading glucosylceramide (GlcCer) within lysosomes. Mutations in this enzyme not only reduce its activity but also cause abnormal accumulation of its substrate, GlcCer [202,203,204]. Research indicates that accumulated GlcCer can self-assemble into twisted ribbon-like amyloid fibrils. These lipid fibrils directly stabilize early-stage αSyn oligomers and accelerate their aggregation process [203,204]. Concurrently, mutant GCase becomes retained in the endoplasmic reticulum due to misfolding or mislocalization to the lysosomal membrane surface. By competitively binding receptors, it blocks CMA, preventing degradation of autophagy substrates including αSyn and further exacerbating protein toxicity [201,203,205].

There exists a significant synergistic effect between αSyn aggregation and lysosomal lipid homeostasis, forming a vicious pathological cycle [178,201,206]. Aggregated αSyn further suppresses residual GCase activity, while GCase deficiency induces mitochondrial complex I dysfunction and energy metabolism failure by disrupting lysosomal–mitochondrial contact points [206,207]. At the subcellular level, αSyn fibrils sequester and consume ESCRT-III complex components required for endosomal membrane repair, leading to lysosomal membrane damage and cytoplasmic escape of pathological seeds, triggering a “second wave” of templated aggregation and intercellular propagation [178,208]. Targeting this pathway, restoring GCase activity via small-molecule chaperones (e.g., ambroxol), or clearing αSyn aggregates using autophagy-targeting chimeras (AUTOTAC) has emerged as key directions in current PD-modifying therapies [205,209].

### 6.3. Multiple Sclerosis or Huntington’s Disease

The pathological core of MS is an autoimmune attack targeting the myelin sheath and its lipid components within the CNS, a process that initiates approximately 7 years before clinical symptoms emerge [210]. Research indicates that Epstein–Barr virus (EBV) infection, in conjunction with the HLA-DR15 haplotype, drives this mechanism: Virus-infected B cells enter brain parenchyma by expressing latent membrane protein 1 (LMP1), directly capture myelin antigens, and present myelin basic protein (MBP) peptides to CD4+ T cells, thereby triggering inflammatory demyelination [211,212]. Additionally, signaling via the formyl peptide receptor 1 (FPR1) on microglia and macrophage surfaces plays a critical role in disease progression by inducing mitochondrial dysfunction, leading to axonal loss and neuronal apoptosis [213].

Myelin lipid metabolites themselves and their transport disorders serve as key initiators in sustaining chronic inflammatory environments. Lipid components within myelin debris, such as lysophosphatidylserine (LysoPS), activate microglia via the GPR34 receptor and induce proinflammatory cytokine expression [214]. Concurrently, glial cells convert myelin’s abundant very long-chain fatty acids (VLCFAs) into sphingosine 1-phosphate (S1P), further inducing neuroinflammation by activating the NF-κB pathway [215]. At the periphery of chronic active lesions in MS, microglia exhibit marked upregulation of lipid metabolism. Deficiency of ATP-binding cassette transporter A1/G1 (ABCA1/G1) leads to the formation of lipid-accumulating phagocytes (i.e., foam cells), amplifying inflammatory signaling and impeding tissue repair [216].

Failure of myelin regeneration is not only influenced by immunosuppression but is also closely associated with phenotypic inactivation of lipid biosynthesis. In MS lesions, oligodendrocytes often exhibit epigenetic silencing, preventing the master lipid metabolic regulator SREBP1/2 from forming normal nuclear aggregates. This impairs cholesterol and lipid biosynthesis, thereby blocking the myelin regeneration process [80]. Furthermore, clusterin secreted by astrocytes inhibits the PI3K-AKT signaling pathway in oligodendrocyte precursor cells (OPCs), further disrupting their differentiation [217].

In other neurodegenerative diseases such as Huntington’s disease (HD), lipid metabolism disorders similarly manifest as homeostatic imbalance. HD models demonstrate impaired secretion of extracellular vesicles regulated by gangliosides (e.g., GM1), leading to intracellular accumulation of mutant huntingtin protein (mHTT); supplementation with GM1 or modulation of lipid metabolic pathways promotes clearance of the pathogenic protein and alleviates neurotoxicity [180]. These findings underscore the decisive role of lipid metabolism reprogramming in diverse neurological disorders, particularly demyelinating lesions in MS.

## 7. Intervention Strategies: Therapeutic Prospects Targeting Lipid Metabolism

### 7.1. Dietary and Nutritional Interventions

The ketogenic diet (KD), as a high-fat, low-carbohydrate metabolic intervention, is emerging as a key strategy for repairing age-related lipid metabolism disorders in the brain. By inducing the body into ketosis, KD generates ketone bodies such as β-hydroxybutyrate (BHB), providing neurons with an alternative energy source that bypasses impaired glucose metabolism pathways [218,219]. In AD models, long-term KD intervention has been shown to significantly improve spatial learning and working memory. This effect involves mechanisms such as restoration of neuronal and synaptic numbers, reduction in amyloid plaque deposition, and suppression of microglial activation [220,221]. Furthermore, KD exerts significant protective effects on BBB by inducing astrocytic A2-type polarization—a neuroprotective state—through inhibition of histone deacetylase 3 (HDAC3) and the NF-κB/NLRP3 inflammasome pathway. This maintains barrier integrity and reduces leukocyte recruitment [222]. In acute neurological injuries such as TBI or SCI, KD and its metabolites (e.g., medium-chain triglycerides, MCT) exhibit multifaceted protective effects, including enhanced mitochondrial bioenergetic efficiency, increased cerebral oxygenation, and reduced oxidative stress [223,224,225].

n-3 polyunsaturated fatty acids (n-3 PUFAs, such as DHA and EPA) play an irreplaceable role in maintaining neuronal membrane integrity and regulating synaptic signaling [226,227]. DHA promotes synaptic spine formation by activating the RXRα signaling pathway and delays cognitive decline by competitively inhibiting Aβ production through upregulation of β-secretase 2 (BACE2) [228,229]. Further studies reveal that n-3 PUFAs and their derivatives (e.g., DPA) exert potent immunomodulatory effects. They remodel microglia from pro-inflammatory M1 to homeostatic or reparative M2 cells by inhibiting NF-κB and MAPK signaling pathways, while activating the neuronal BDNF/TrkB/CREB signaling axis to suppress apoptosis [52,230,231]. Moreover, n-3 PUFAs enhance hippocampal insulin sensitivity by optimizing the IRS-1/AKT/GLUT4 pathway, thereby disrupting the vicious cycle of “metabolic dysfunction–neuroinflammation–protein pathology”. This is particularly crucial for patients with metabolic syndrome or type 2 diabetes [232,233].

The clinical translation efficiency of lipid interventions is highly dependent on their molecular carrier form and systemic metabolic context. Compared to traditional triglyceride forms, DHA bound to lysophosphatidylcholine (LPC) can more efficiently cross the BBB via the Mfsd2a transporter, increasing DHA abundance in the brain and retina by several-fold [234,235]. Concurrently, the gut microbiota is recognized as a critical bridge mediating dietary lipid effects on brain health. Supplementation with n-3 PUFAs indirectly alleviates neuroinflammation by improving gut microbiota composition and reducing serum LPS levels [236,237,238]. Combining specific nutrient supplementation (e.g., ALA-rich flaxseed oil or algae oil) with regular aerobic exercise may produce significant synergistic effects in improving mitochondrial resilience, enhancing neurogenesis, and elevating quality of life in the elderly population [230,239,240].

### 7.2. Pharmacological Intervention

LXR/RXR agonists, as key candidate drugs for regulating brain lipid homeostasis, demonstrate significant neuroprotective potential by promoting cholesterol efflux in the brain. LXR agonists (such as LXR623, 22-ketocholesterol, and novel non-lipogenic ABCA1 inducers) significantly upregulate ABCA1 expression and enhance ApoE particle lipidation, thereby alleviating neuronal degeneration caused by lipid metabolism defects [241,242,243]. In models of AD and hereditary spastic paraplegia (HSP), LXR activation has been demonstrated to effectively rescue synaptic function, reduce tau pathology, and suppress pro-inflammatory activation of microglia [241,244]. Specifically, in TREM2-deficient microglia, LXR agonists restore dysfunctional glial cells to homeostasis by promoting cholesterol ester clearance, offering a precisely targeted strategy to correct lipid accumulation in the brain [197,244].

The use of statins in neurological disorders has been accompanied by long-standing controversy, with the focus on whether their peripheral lipid-lowering effects can effectively translate into central nervous system protection. Epidemiological and retrospective cohort studies indicate that statin use is strongly associated with reduced mortality, preserved cognitive function, and decreased risk of Parkinsonian syndromes in Alzheimer’s disease patients [245,246]. However, Mendelian randomization studies also suggest that genetic suppression of HMG-CoA reductase may exert potential adverse effects on certain cognitive dimensions (e.g., reaction speed), underscoring the importance of precision medication [247]. The neuroprotective effects of statins may stem from multifaceted mechanisms beyond lipid-lowering, including reduced MMP-9 activity, improved cerebral blood flow, inhibition of microglial activation, and action via PACAP-related pharmacological pathways [248,249]. Notably, the physicochemical properties of different statins determine their efficacy: hydrophobic statins (e.g., simvastatin) can directly cross the blood–brain barrier and alter neuronal membrane microviscosity and lipid order, whereas hydrophilic statins (e.g., pravastatin) lack such direct membrane-modulating effects [250].

**Figure 6:**
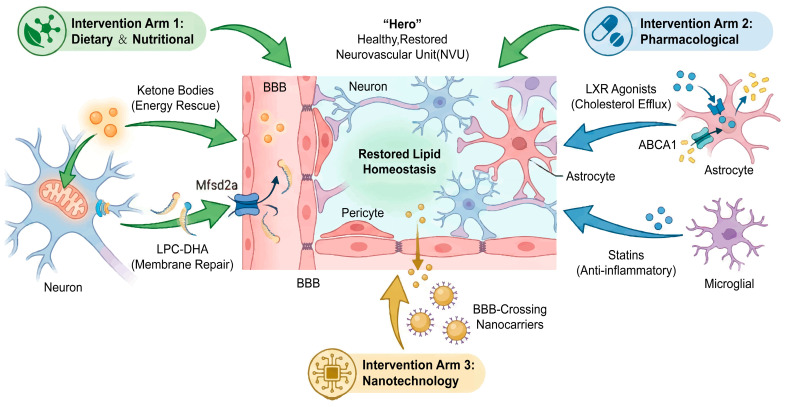
Multidimensional intervention strategies: reshaping brain lipid homeostasis. This schematic illustrates a multipronged therapeutic approach targeting the neurovascular unit (NVU) to rescue lipid dysmetabolism and neuroinflammation. (**Left**) Dietary and nutritional interventions leverage specific metabolic substrates to support neuronal health: ketone bodies provide an alternative bioenergetic substrate to rescue neuronal mitochondrial function (energy rescue), while targeted supplementation with LPC-DHA facilitates structural membrane repair by actively crossing the blood–brain barrier (BBB) endothelium via the Mfsd2a transporter. (**Right**) Pharmacological interventions modulate glial cell lipid handling and activation states. Liver X receptor (LXR) agonists target astrocytes to upregulate ABCA1 expression, thereby promoting physiological cholesterol efflux and preventing lipotoxicity. Concurrently, statins exert targeted anti-inflammatory effects by attenuating pathological microglial activation. (**Bottom**) Nanotechnology platforms utilize engineered, BBB-crossing nanocarriers to effectively bypass the restrictive endothelial barrier and deliver therapeutic cargo directly into the brain parenchyma. The convergence of these distinct modalities (indicated by directional arrows) synergistically promotes the structural and functional recovery of the cellular components, ultimately culminating in a state of restored lipid homeostasis within the central NVU. ABCA1, ATP-binding cassette transporter A1; BBB, blood–brain barrier; LPC-DHA, lysophosphatidylcholine-docosahexaenoic acid; LXR, liver X receptor; Mfsd2a, major facilitator superfamily domain containing 2A; NVU, neurovascular unit.

Pharmacological interventions targeting lipodrop formation, particularly by inhibiting cholesterol esterification through ACAT1 (also known as SOAT1) enzyme suppression, are emerging as a novel approach to enhance cellular protein homeostasis and autophagy. ACAT1 catalyzes the conversion of intracellular free cholesterol into cholesterol esters stored within lipid droplets. Its inhibitors enhance ABCA1-mediated cholesterol efflux by increasing the free cholesterol pool, thereby mitigating lipotoxicity-induced damage [251]. In pathological contexts where lipid turnover is impaired, such as TREM2 deficiency, ACAT1 inhibitors have been shown to effectively reverse pathological accumulation of cholesterol esters in glial cells, restoring their lysosomal degradation capacity and autophagic flux [197]. This strategy not only shows potential for reducing pathological burden in AD models but also provides insights for systemic diseases involving lipid metabolism disorders, such as diabetic nephropathy [251]. Furthermore, novel carrier systems—like the donepezil-simvastatin nanomodulator—aim to overcome blood–brain barrier barriers, enabling efficient drug accumulation in the central nervous system to remodel the lesion microenvironment at the etiological level [252] (Figure 6).

## 8. Conclusions and Perspectives

As the organ with the highest lipid content in the human body, the brain’s structural integrity and signal transduction are highly dependent on the precise regulation of lipid homeostasis. Reviewing the entire text, lipid metabolic reprogramming plays a central driving role in the onset and progression of neurodegenerative diseases (NDDs). This is not merely a pathological concomitant phenomenon, but rather a key “trigger” linking physiological aging to pathological neurodegeneration. During natural aging, the brain undergoes “lipid drift” characterized by decreased membrane fluidity, impaired cholesterol transport, and loss of PUFAs [39,46]. When this accumulated metabolic stress interacts with genetic risk factors (e.g., APOE4), environmental toxins, or chronic inflammation, intracellular adaptive compensatory mechanisms collapse, triggering lethal lipid metabolic reprogramming.

This process manifests primarily as altered metabolic heterogeneity in glial cells. For instance, microglia transition to an LDAM due to impaired lipid degradation, thereby losing phagocytic function and exacerbating inflammation [122]. Concurrently, impaired FAO in astrocytes disrupts metabolic support for neurons, leading to lipotoxicity injury [136]. Furthermore, the collapse of subcellular organelle function is a critical component: lysosomes undergo membrane permeabilization (LMP) due to lipofuscin and cholesterol accumulation, blocking autophagic flux [62], while mitochondria lose membrane potential under ceramide attack, triggering energy depletion and apoptosis [140].

**Figure 7:**
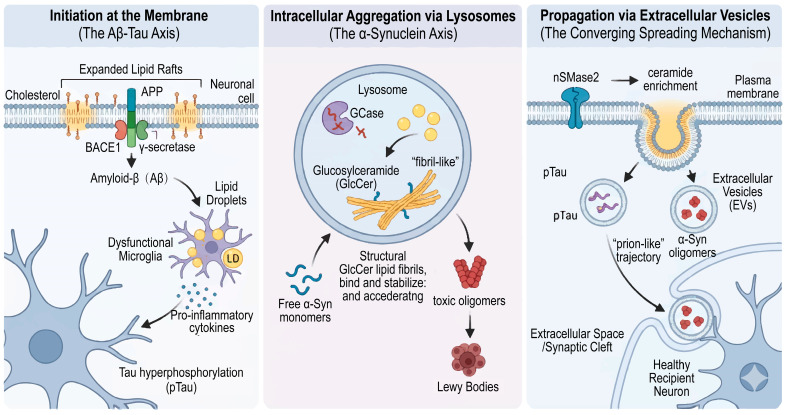
Converging lipid-driven pathways in the pathogenesis and propagation of Aβ, pTau, and α-synuclein. (**Left**) At the neuronal membrane, expanded cholesterol-enriched lipid rafts facilitate amyloidogenic processing of the amyloid precursor protein (APP). Cleavage by BACE1 and γ-secretase releases amyloid-β (Aβ) peptides, which subsequently induce lipid droplet (LD) accumulation and dysfunction in adjacent microglia. These dysfunctional microglia secrete pro-inflammatory cytokines that trigger downstream aberrant tau phosphorylation (pTau) in neurons, leading to modifications at specific pathological epitopes rather than physiological sites. (**Middle**) Within the intracellular compartment, dysfunctional GCase leads to the lysosomal accumulation of glucosylceramide (GlcCer). These accumulated lipids form physical “fibril-like” structural scaffolds that bind and stabilize free α-synuclein (α-Syn) monomers, accelerating their pathological conversion into toxic oligomers and, ultimately, Lewy bodies. (**Right**) The propagation phase converges on extracellular vesicle (EV) pathways. Activation of nSMase2 at the plasma membrane causes localized ceramide enrichment, driving the outward budding of EVs. These ceramide-enriched EVs package pathogenic pTau and α-Syn oligomers, illustrating the early-stage active clearance and propagation mechanism before tangle-induced cell lysis. The directional trajectory highlights a shared “prion-like” trans-cellular propagation mechanism, where EVs traverse the extracellular space and synaptic cleft to fuse with healthy recipient neurons, thereby disseminating the pathology. Aβ, amyloid-β; APP, amyloid precursor protein; BACE1, β-site APP cleaving enzyme 1; EV, extracellular vesicle; GCase, glucocerebrosidase; GlcCer, glucosylceramide; LD, lipid droplet; nSMase2, neutral sphingomyelinase 2; pTau, pathologically phosphorylated tau; α-Syn, α-synuclein.

Lipid dyshomeostasis serves as a fundamental nexus driving the generation; aggregation; and trans-cellular propagation of key pathogenic proteins, including amyloid-beta (Aβ), pathologically phosphorylated tau (pTau)—characterized not necessarily by an absolute increase in phosphate groups, but by aberrant phosphorylation at disease-associated amino acid residues [253,254]—and α-synuclein (α-Syn). Based on current evidence, a generalized, interconnected framework can be proposed. Initially, pathological cholesterol accumulation and the physical expansion of lipid rafts provide a critical structural platform for APP processing, initiating the Aβ generation cascade [96]. The resultant Aβ accumulation further acts as an upstream stressor, inducing microglial lipid droplet formation and triggering inflammatory factor release [122], which subsequently exacerbates tau phosphorylation. Parallel to tau pathology, α-Syn aggregation is profoundly governed by lipid membrane interactions; specifically, lysosomal glucocerebrosidase (GCase) dysfunction leads to glucosylceramide (GlcCer) accumulation, forming lipid fibrils that directly stabilize early α-Syn oligomers. Crucially, the progression of both AD and PD converges on a shared exosome-mediated propagation mechanism. Dysregulated sphingolipid metabolism, characterized by neutral sphingomyelinase 2 (nSMase2) activation and ceramide enrichment in extracellular vesicles (EVs), not only drives the widespread release of pTau but also serves as a pathogenic carrier that interacts with wild-type α-Syn, lowering its aggregation threshold and accelerating its “prion-like” spreading across neural networks, as shown in Figure 7.

However, it is imperative to acknowledge that this proposed sequence of events may be significantly altered under different physiological conditions or across distinct types of neurodegenerative diseases. The temporal dynamics of lipid–protein interactions likely exhibit considerable heterogeneity depending on the specific local microenvironment and disease stage. While current models provide a robust theoretical foundation, further rigorous longitudinal and in vivo mechanistic studies are necessary to firmly prove the causality of these specific lipid-related events.

Ultimately, alterations in lipid raft architecture and dysregulated exosome secretion promote Aβ production and the trans-regional spread of Tau and α-Syn, establishing a vicious “metabolic–immune–neurotoxic” feedback loop that disintegrates neural networks [96].

Current neurodegenerative disease diagnosis often relies on late-stage protein pathological markers, whereas alterations in lipid metabolism typically occur years or even decades before clinical symptoms and protein aggregation emerge. Therefore, high-throughput lipidomics based on mass spectrometry technology holds promise for redefining the paradigm of early disease diagnosis. Establishing peripheral blood lipid fingerprints is particularly crucial. Specific blood lipids, such as plasma ceramides (especially the C16:0/C24:0 ratio), LPC, and specific acylcarnitine profiles, have been demonstrated to sensitively reflect the metabolic state of the central nervous system. These are poised to become non-invasive liquid biopsy biomarkers for large-scale population risk screening [38,169]. Concurrently, lipid components in CSF and brain-derived exosomes more directly reflect alterations in the brain microenvironment. For instance, CSF sulfatide depletion is considered an early hallmark of AD [27], while changes in exosomal membrane lipids predict the risk of pathogenic protein spread [255]. Future research should focus on integrating lipidomics with proteomics and genomics data to construct “lipid–protein–gene” interaction networks. This approach aims to identify disease subtypes with specific lipid metabolic signatures, enabling precise stratified diagnosis [1].

Despite the promising therapeutic prospects of targeting lipid metabolism, the presence of the BBB poses significant challenges for brain-specific lipid regulation. Future intervention strategies must focus on overcoming BBB limitations to achieve targeted delivery of drugs or nutrients. First, leveraging endogenous transport channels represents an efficient strategy. Given that the Mfsd2a transporter on the BBB can specifically transport LPC-DHA into the brain, developing LPC-based lipid precursor drugs or nutritional supplements offers an effective pathway to bypass BBB limitations and replenish essential fatty acids within the brain [10,235]. Second, The advancement of nanodelivery systems enables the delivery of hydrophobic drugs. Utilizing biomimetic nanotechnologies (e.g., exosomes and liposomes) or functionalized nanocarriers (e.g., transferrin receptor-modified nanoparticles) can transport LXR agonists or ACAT1 inhibitors across the BBB. For instance, carrier-free nanomodulators have been demonstrated to directly enter the brain via intranasal administration, reshaping the microenvironment at the lesion site [252]. Finally, cell-specific targeting is crucial for enhancing efficacy and reducing side effects. Future drug development should focus on distinguishing between “good” and “bad” lipid pools, such as specifically targeting lipodegradation in microglia (e.g., by activating the TRPV1-PKM2 axis) without interfering with normal neuronal lipid synthesis [256]. In summary, as our understanding of the brain’s lipid metabolism landscape deepens, we are entering a new era of neuroprotection centered on “restoring lipid homeostasis”.

## Data Availability

No new data were created or analyzed in this study. Data sharing is not applicable to this article.
